# Editorial for the Special Issue on Analysis, Design and Fabrication of Micromixers

**DOI:** 10.3390/mi12050533

**Published:** 2021-05-07

**Authors:** Kwang-Yong Kim

**Affiliations:** Department of Mechanical Engineering, Inha University, Incheon 22212, Korea; kykim@inha.ac.kr; Tel.: +032-860-7317

During the last couple of decades, there have been rapid developments in analysis, design, and fabrication of micromixers. Micromixers are essential components of micro-total analysis system (μ-TAS) and lab-on-a-chip, and have various chemical and biological applications [1]. Due to the small scale, flows of the liquid samples in micromixers are laminar, and thus have a difficulty in mixing. Therefore, efficient mixing is a critical goal in the design of micromixers. A variety of passive or active designs are used to enhance the mixing. Design optimization is an efficient tool to maximize the mixing by optimizing the geometric and/or operating parameters using systematic algorithms. Both numerical and experimental methods have been used to analyze the flow and mixing in micromixers. However, due to the micro scale, quantitative experimental measurement of mixing is quite limited in micromixers, and special techniques are also required for the fabrication.

The current special issue includes 12 research papers [2,3,4,5,6,7,8,9,10,11,12,13]. The topics of the papers are focused on design of micromixers [2,3,4,5,6,7,9,12,13], fabrication [8,9,12], and analysis [11]. Some of them propose novel micromixer designs [2,3,5,6], which include a slight modification of an existing micromixer [6]. Most of them deal with passive micromixers, but two papers [5,11] report the studies on electrokinetic micromixers. Fully three-dimensional (3D) micromixers were investigated in some cases [2,3,4,13]. Hossain et al. [4] applied optimization techniques to the design of a 3D micromixer. Raza et al. [13] performed a review of recently developed passive micromixers and a comparative analysis of 10 typical micromixers. Martinez-Lopez et al. [8], Wang et al. [9], and Sabotin et al. [12] focused on the fabrication techniques for micromixers, and two of them [8,9] used 3D print techniques.

The review paper of Raza et al. [13] has a unique feature that is different from other reviews on micromixers. They classified passive micromixer designs developed so far into five types: 2D designs using serpentine, spiral, curved helical channels, 2D designs with split-and-recombination (SAR) structures, 3D design with serpentine and/or SAR structures, 3D design with patterned grooves, and 3D designs with SAR two-layer crossing channels. Along with a general review of micromixers in each type, they performed a quantitative comparison among 10 representative micromixers selected from the five types with their own calculations. For this comparison, they calculated the mixing indexes and pressure drops of theses micromixers under the same axial length and Reynolds numbers. Eight Reynolds numbers in a range of Re = 0.01–120 were tested, which have been selected from three ranges: low-Re (Re < 1), intermediate-Re (1 < Re < 40), and high-Re (Re > 40) ranges. Even through a number of micromixer designs have been developed, there has not been any quantitative comparison of these micromixers under the same operating conditions. Therefore, this review report is expected to contribute to the selection of effective micromixers for specific applications.

Martinez-Lopez et al. [8] characterized photopolymers and stereolithography processes for manufacturing 3D print molds and polydimethylsiloxane castings of micromixers. Validation of the soft tooling approach was performed for an asymmetric SAR micromixer with different cross sections under various flow conditions (10 < Re < 70). Wang et al. [9] designed a jet mixer with arrays of micro-nozzles and fabricated it using 3D printing technology. The design has two opposite arrays of micro-nozzles to enhance the mixing performance with chaotic advection. The mixing efficiency of the mixer was measured experimentally and analyzed comparatively with a conventional Y-shaped micromixer. Sabotin et al. [12] constructed a simple technical model of micro electrical discharge machining (EDM) milling. They performed an experiment by machining different microgrooves into corrosive-resistant steel and tested the following parameters: material removal rate, electrode dressing time, machining strategy, electrode wear, and electrode wear control time. The technical model was demonstrated through a bottom grooved micromixers (BGM) design. The mixing performances of different BGM designs were evaluated numerically to find an optimum design.

Different methods have been applied to the analysis of mixing. Most of the works used only computational fluid dynamics (CFD) for the analysis [2,3,4,5,6,10,13]. However, some of the works employed experimental analysis [8,9] or both experimental and CFD analyses [7,12]. On the other hand, Kim et al. [11] performed a theoretical analysis of an electromagnetic micromixer and compared the results with experimental measurements. They suggested the physical foundation for the electromagnetic coupling in the micromixer, and derived a correlation between the mechanical velocity and electrical driving point impedance. Exceptionally, Granados-Ortiz and Ortega-Casanova [10] analyzed the thermal mixing between two parallel laminar flows with different temperatures in a simple 2D channel with an obstacle located at the center. They performed unsteady 2D Navier-Stokes analysis to analyze the mixing caused by the vortex shedding behind the obstacle.

I would like to express my appreciation to all the experts who contributed to this special issue. I also thank all the reviewers of the submitted papers. This special issue will be continued as Analysis, Design and Fabrication of Micromixers II. I hope for further up-to-date technologies of micromixers to be reported in the next special issue.
